# *Plasmodium ovale wallikeri* in Western Lowland Gorillas and Humans, Central African Republic

**DOI:** 10.3201/eid2408.180010

**Published:** 2018-08

**Authors:** Mwanahamisi I. Mapua, Hans-Peter Fuehrer, Klára J. Petrželková, Angelique Todd, Harald Noedl, Moneeb A. Qablan, David Modrý

**Affiliations:** University of Veterinary and Pharmaceutical Sciences, Brno, Czech Republic (M.I. Mapua, M.A. Qablan, D. Modrý);; University of Veterinary Medicine, Vienna, Austria (H.-P. Fuehrer);; Czech Academy of Sciences, Brno (K.J. Petrželková);; Liberec Zoo, Liberec, Czech Republic (K.J. Petrželková);; Czech Academy of Sciences, České Budějovice, Czech Republic (K.J. Petrželková, D. Modrý);; World Wildlife Fund, Bangui, Central African Republic (A. Todd);; Medical University of Vienna (H. Noedl);; United Arab Emirates University, Al Ain, United Arab Emirates (M.A. Qablan)

**Keywords:** western lowland gorillas, malaria, *Plasmodium ovale wallikeri*, Central African Republic, parasites, zoonoses

## Abstract

Human malaria parasites have rarely been reported from free-ranging great apes. Our study confirms the presence of the human malaria parasite *Plasmodium ovale wallikeri* in western lowland gorillas and humans in Dzanga Sangha Protected Areas, Central African Republic, and discusses implications for malaria epidemiology.

The transmission of infectious diseases between wild animals and humans is a dynamic process in which wildlife populations have been a major source of zoonotic diseases. Six human malaria parasite species have been recognized during the past decade: *Plasmodium falciparum*, *P. vivax*, *P. malariae*, *P. knowlesi*, *P. ovale curtisi*, and *P. ovale wallikeri*. The role of human malaria parasites as zoonotic agents in free-ranging great apes remains unclear. Various *Plasmodium* species have been documented in great apes by using molecular data, including *P. praefalciparum*, *P. adleri*, and *P. blacklocki* in western lowland gorillas (*Gorilla gorilla gorilla*) (*1*). Moreover, parasites closely related to human malaria parasites (e.g., *P. ovale*–like and *P. vivax*–like) have been identified in other free-ranging great apes (*2*). Although contact between free-ranging gorillas and humans has increased steadily, no shared malaria parasites have previously been reported in humans and gorillas. A recent study of western lowland gorillas revealed a unique case of *P. ovale* infection, based on amplification of the mitochondrial cytochrome b (*cytb*) gene from gorilla fecal samples (*1*). To clarify the implications of possible co-circulation of malaria parasites in humans and gorillas, we reevaluated human blood and western lowland gorilla fecal samples from the previous study using state-of-the-art molecular tools.

Dzanga Sangha Protected Areas is located in southwestern Central African Republic, bordering the Democratic Republic of the Congo in the east and Cameroon in the west. We collected 131 gorilla fecal samples during August–October 2012 from 2 habituated western lowland gorilla groups and from several unhabituated gorillas around the Primate Habituation Programme camps of Mongambe and Bai Hakou. We collected human blood samples in 2012 as part of a health monitoring program for National Park and Primate Habituation Project employees. We received 95 blood samples from asymptomatic participants.

We performed PCR within the nuclear 18S small subunit ribosomal RNA gene, as previously described (*3*,*4*). This gene is the most commonly used for diagnosis of *P. falciparum*, *P. vivax*, *P. malariae*, *P. ovale* (primers rOVA1WC/rOVA2WC), *P. ovale curtisi* (rOVA1/rOVA2), *P. ovale wallikeri* (rOVA1v/rOVA2v), and *P. knowlesi* in humans. We amplified parts of the mt *cytb* gene to obtain ≈939-bp fragments (*1*). We also analyzed a shorter fragment of ≈480 bp with hemosporidian-specific primers (*5*). We used a hemosporidian-specific nested PCR performed within the mitochondrial cytochrome c oxidase subunit 1 gene (primers: cox1a/cox1b and cox1c/cox1d) to amplify a 964-bp fragment (*6*). For further analysis of *P. ovale*, we used the primers Porbp2TMfwd/Porbp2TMrev, binding a 120-bp fragment of the nuclear *P. ovale* reticulocyte binding protein 2 gene (*7*).

Overall, 23 out of 95 (24.2%; 95% CI 16.7%–33.7%) asymptomatic human samples had positive results in *Plasmodium*-specific PCRs. Species-specific analysis revealed *P. falciparum* monoinfections in 19 cases (20%; 95% CI 13.2%–29.1%) and *P. ovale wallikeri* in 2 cases (2.1%; 95% CI 0.6%–7.3%). We found a mixed infection of *P. falciparum* and *P. malariae* (2.1%; 95% CI 0.6%–7.3%) in 2 samples. In the previous study, 42 of 131 fecal samples from western lowland gorillas tested positive for *Plasmodium* parasites, namely *P. adleri* (n = 21), *P. praefalciparum* (n = 9), and *P. blacklocki* (n = 7), but also *P. ovale*–like (n = 1) and *P. vivax*–like (n = 3) (*1*). In this study, reevaluating the gorilla fecal DNA extracts for human malaria parasites confirmed 1 sample from a habituated individual as positive for *P. ovale wallikeri*. Identical sequences were obtained in mt *cytb* (GenBank accession no. KJ930413) and mt *cox*1 (GenBank accession nos. MG251662, MG251663) with the gorilla *P. ovale wallikeri* isolate and 1 of the human isolates (Figure). The analysis of the nuclear small subunit rRNA and the *porbp*2 gene gave positive results only for the human isolate (GenBank accession nos. MG251661, MG255222).

**Figure:**
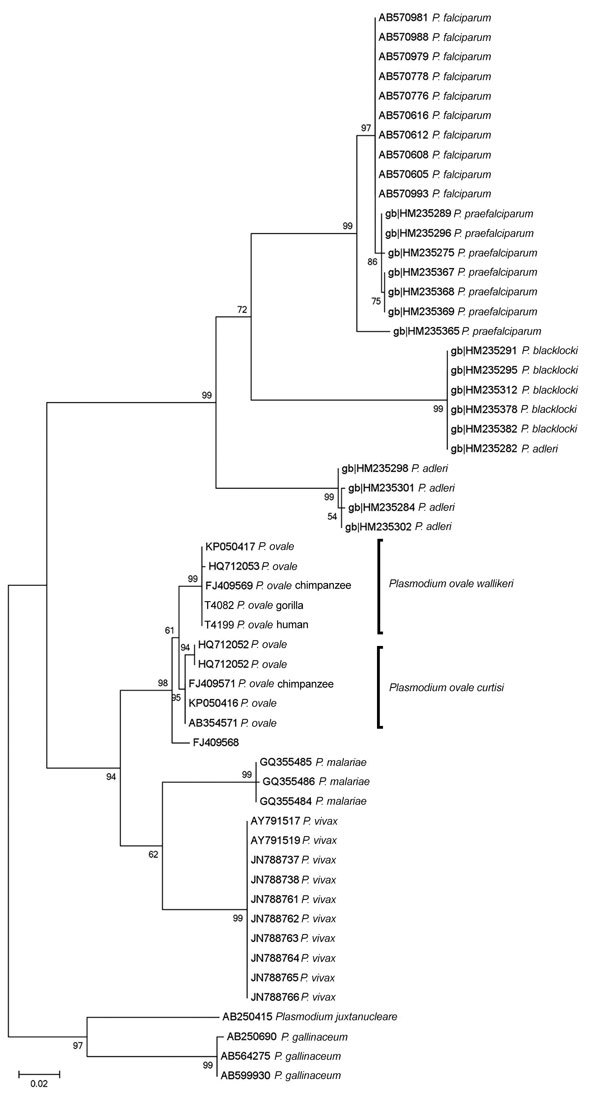
Maximum-likelihood phylogenetic trees of cytochrome C oxidase subunit 1 (*cox*1) gene (656-bp) sequences from African great apes and human *Plasmodium* spp. reference strains. GenBank accession numbers are indicated. Scale bar represents nucleotide substitutions per site.

In this study, we found the human malaria parasite *P. ovale wallikeri* in both asymptomatic humans and western lowland gorillas in Dzanga Sangha Protected Areas. Molecular analysis revealed that the genotype of the *P. ovale wallikeri* DNA found in a gorilla was genetically identical to that of a human isolate within the mt *cytb* and mt *cox*1 genes, indicating potential human–ape transmission. Analysis of nuclear genes failed in gorilla feces; thus, it remains unclear which parasite stages can be detected in feces (*8*). Although the mt *cytb* and mt *cox*1 genes are not the best-suited genes for genotyping human malaria parasites because of their homogeneity, these genes allow clear species discrimination from *P. ovale*–like parasites found in, for example, chimpanzees in Côte d’Ivoire (*9*) and the Democratic Republic of the Congo (*10*), which have never been reported in humans. This finding in a western lowland gorilla corroborates a finding of *P. ovale* in a chimpanzee from Cameroon (*6*) with a sequence identical to *P. ovale wallikeri*. However, further studies are required to evaluate the role of great apes as reservoirs of human malaria parasites and vice versa, and the implications of these findings for malaria epidemiology.
